# Soil legacy data rescue via GlobalSoilMap and other international and national initiatives

**DOI:** 10.1016/j.grj.2017.06.001

**Published:** 2017-12

**Authors:** Dominique Arrouays, Johan G.B. Leenaars, Anne C. Richer-de-Forges, Koushik Adhikari, Cristiano Ballabio, Mogens Greve, Mike Grundy, Eliseo Guerrero, Jon Hempel, Tomislav Hengl, Gerard Heuvelink, Niels Batjes, Eloi Carvalho, Alfred Hartemink, Alan Hewitt, Suk-Young Hong, Pavel Krasilnikov, Philippe Lagacherie, Glen Lelyk, Zamir Libohova, Allan Lilly, Alex McBratney, Neil McKenzie, Gustavo M. Vasquez, Vera Leatitia Mulder, Budiman Minasny, Montanarella Luca, Inakwu Odeh, Jose Padarian, Laura Poggio, Pierre Roudier, Nicolas Saby, Igor Savin, Ross Searle, Vladimir Solbovoy, James Thompson, Scott Smith, Yiyi Sulaeman, Ruxandra Vintila, Raphael Viscarra Rossel, Peter Wilson, Gan-Lin Zhang, Martine Swerts, Katrien Oorts, Aldis Karklins, Liu Feng, Alexandro R. Ibelles Navarro, Arkadiy Levin, Tetiana Laktionova, Martin Dell’Acqua, Nopmanee Suvannang, Waew Ruam, Jagdish Prasad, Nitin Patil, Stjepan Husnjak, Laszlo Pasztor, Joop Okx, Stephen Hallet, Caroline Keay, Timothy Farewell, Harri Lilja, Jerome Juilleret, Simone Marx, Yusuke Takata, Yagi Kazuyuki, Nicolas Mansuy, Panos Panagos, Mark Van Liedekerke, Rastislav Skalsky, Jaroslava Sobocka, Josef Kobza, Kamran Eftekhari, Seyed Kacem Alavipanah, Rachid Moussadek, Mohamed Badraoui, Mayesse Da Silva, Garry Paterson, Maria da Conceicao Gonsalves, Sid Theocharopoulos, Martin Yemefack, Silatsa Tedou, Borut Vrscaj, Urs Grob, Josef Kozak, Lubos Boruvka, Endre Dobos, Miguel Taboada, Lucas Moretti, Dario Rodriguez

**Affiliations:** 1 INRA, InfoSol Unit, 45075 Orleans, France; 2 ISRIC - World Soil Information, PO Box 353, 6700 AJ, Wageningen, The Netherlands; 3 Department of Agroecology, Faculty of science and technology, Aarhus University, Tjele, Denmark; 4 Departamento de Suelos, National Agrarian University, La Molina, Peru; 5 European Commission - DG JRC, Via E. Fermi, 2749, 21027 Ispra (VA), Italy; 6 CSIRO, Australia; 7 National Institute of Statistics and Geography, INEGI, Aguascalientes, Mexico; 8 Retired. US department of Agriculture, Natural Resources Conservation Services, Lincoln, Nebraska, USA; 9 University of Wisconsin, Department of Soil Science, Madison, USA; 10 Landcare research, Manaaki Whenua, New-Zealand; 11 Department of Agricultural Environment, National Academy of Agricultural Science, Suwon 441-707, Korea; 12 Eurasian Center for Food Security, Lomonosov Moscow State University, Moscow, Russia; 13 INRA-IRD-Supagro, UMR Lisah, Montpellier, France; 14 Agriculture and Agri-Food Canada, Ottawa, Canada; 15 US department of Agriculture, Natural Resources Conservation Services, Lincoln Nebraska, USA; 16 The James Hutton Institute, Craigiebuckler, Aberde **l**and, UK; 17 University of Sydney, Sydney, Australia; 18 Embrapa Solos, Rio de janeiro, Brazil; 19 Biogeochemistry and Earth System Modelling, Sciences de la Terre et de l’Environnement, Universite Libre de Bruxelles, Bruxelles, Belgium; 20 Dokutchaev Soil Science Institute, Moscow, Russia; 21 West Virginia University, West Virginia, USA; 22 Indonesian Center for Agricultural Land Resource Research and Development, Bogor, West Javal Indonesia; 23 National Research and Development Institute for Soil Science, Agro-chemistry and Environment (ICPA), Bucharest, Romania; 24 State Key Laboratory of Soil and Sustainable Agriculture, Institute of Soil Science, Chinese Academy of Sciences, Nanjing, China; 25 Land and Soil Protection Service, Flemish Government, Brussels, Belgium; 26 Latvia Agricultural University, Jelgava, Latvia; 27 National Scientific Center, Institute for Soil Science and Agrochemistry Research, Kharkiv, Ukraine; 28 Sistema de Informacion Geografica, Direccion General de Recursos Naturales, Ministerio de Ganaderia Agricultura y Pesca, Montevideo, Uruguay; 29 Land Development Department, Land Development Department, Ladyao, Chatuchak, Bangkok, Thailand; 30 National Bureau of Soil Survey and Land Use Planning (NBSSLUP) Indian Council of Agricultural Research (ICAR), Nagpur, Maharashtra, India; 31 Faculty of Agriculture, Deptartment of Soil Science, Zagreb, Croatia; 32 Research Institute for Soil Science and Agricultural Chemistry (RISSAC) of the. Hungarian Academy of Sciences, Budapest, Hungary; 33 Alterra - Wageningen UR, 6700 AA, Wageningen, The Netherlands; 34 Cranfield University, Soils and Agrifood Institute, NSRI, United Kingdc; 35 MTT, Plant Production Research, Soil and Plant Nutrition, Jokioinen, Finland; 36 Luxembourg Institute of Science and Technology, Belvaux, Grand Duchy of Luxembourg; 37 Institute for Agro-Environmental Sciences, NARO, Tsukuba, Ibaraki, Japan; 38 Nat Resources Canada, Canadian Forest Serv, Ste Foy, Quebec City, Canada; 39 Soil Science and Conservation Research Institute, Gagarinova 10, Bratislava, Slovakia; 40 Soil genesis and classification department, Soil and water research institute, Karaj, I. R. Iran; 41 INRA, Rabat, Morocco; 42 International Center for Tropical Agriculture (CIAT) - Headquarters and Regional Office for Latin America and the Caribbean, Cali, Colombia; 43 ARC-Institute for Soil, Climate and Water, Pretoria, South Africa; 44 Instituto Nacional de Investigagao Agraria e Veterinaria, Laboratorio de Solos de Oeiras, Oeiras, Portugal; 45 Soil Science Institute of Athens, NAGREF, Athens, Greece; 46 International Institute of Tropical Agriculture (IITA) and Institute of Agricultural Research for Development (IRAD), Yaounde, Cameroon; 47 Agricultural Institute of Slovenia, Ljubljana, Slovenia; 48 NABODAT, Agroscope, Institut fur Nachhaltigkeitswissenschaft, INH, Zurich, Switzerland; 49 Faculty of Agrobiology, Czech University of Life Sciences, Prague, Czech Republic; 50 University of Miskolc, geography Institute, Miskolc-Egyetemvaros, Hungary; 51 Institute of Soils, INTA, Buenos Aires, Argentina; 52 People’s Friendship University of Russia (RUDN University), Moscow, Russia

**Keywords:** Soil data rescue, legacy data, GlobalSoilMap

## Abstract

Legacy soil data have been produced over 70 years in nearly all countries of the world. Unfortunately, data, information and knowledge are still currently fragmented and at risk of getting lost if they remain in a paper format. To process this legacy data into consistent, spatially explicit and continuous global soil information, data are being rescued and compiled into databases. Thousands of soil survey reports and maps have been scanned and made available online. The soil profile data reported by these data sources have been captured and compiled into databases. The total number of soil profiles rescued in the selected countries is about 800,000. Currently, data for 117, 000 profiles are compiled and harmonized according to GlobalSoilMap specifications in a world level database (WoSIS). The results presented at the country level are likely to be an underestimate. The majority of soil data is still not rescued and this effort should be pursued. The data have been used to produce soil property maps. We discuss the pro and cons of top-down and bottom-up approaches to produce such maps and we stress their complementarity. We give examples of success stories. The first global soil property maps using rescued data were produced by a top-down approach and were released at a limited resolution of 1km in 2014, followed by an update at a resolution of 250m in 2017. By the end of 2020, we aim to deliver the first worldwide product that fully meets the GlobalSoilMap specifications.

## Introduction

Unprecedented demands are being placed on the world’s soil resources [1–5]. Responding to these challenging demands requires relevant, reliable and applicable information [6–7]. Unfortunately, data, information and knowledge of the world’s soil resources are currently fragmented and even at risk of being lost or forgotten, due to the costs involved with maintaining analogue paper based soil data holdings and archives and the physical deterioration or disintegration of these paper based sources, especially in tropical conditions, together with the risk of the storage buildings (fire, storm, war...). If this were to happen, it would be a disaster not only because soil data are central to many of the major global issues the world is facing [3–5], but also because tremendous resources went into the efforts to collect and analyze these data and comparable future soil data collection would certainly be cost prohibitive in many countries and not justifiable without first having made optimal use of earlier collected data.. Therefore, existing legacy and heritage soil survey data holdings across the world are being rescued, compiled and processed into a common, consistent and geographically contiguous applicable dataset of relevant soil properties covering the planet’s land surface. The legacy soil data holdings, including tens of thousands of published soil reports and soil maps, have been produced over 70 years by nearly all countries and numerous institutions using different procedures, laboratory methods, standards, scales, taxonomic classification systems and geo-referencing systems. They represent a true myriad of primary data (millions of soil profile point observations) and secondary data (derived properties and conventional soil polygon maps).

The GlobalSoilMap project [6–8] provides a collaborative scientific framework to process this legacy soil data into consistent, spatially explicit and continuous global soil information, freely accessible and in a gridded format at a high resolution, thus being both globally complete and locally accurate and thus relevant from global to local applications. The targeted information includes predicted values of selected key soil properties at 6 standard depth intervals (0-5; 5-15; 15-30; 30-60; 60-100; and 100-200 cm), at a global scale on a 3 arc-second support grid (approximately 90x90 m) along with their uncertainties. The key primary soil properties include clay, silt and sand content, coarse elements, pH, soil organic carbon (SOC), effective cation exchange capacity (ECEC) and soil depth to bedrock and effective root zone depth. Additional key properties include bulk density, plant-available water holding capacity and electrical conductivity. The predictions and estimations are generated using state-of-the- art Digital Soil Mapping techniques [9-%^10^].

Hence, obtaining the required amount of primary soil data to produce the above mentioned products, by sampling through new soil surveys, would entail astronomic costs. In comparison, it is relatively cost efficient to utilize existing soil data and make them available and suitable for use. However, one of the major challenges is to integrate the best available legacy data from various local and national sources. This challenge became vital to the GlobalSoilMap project as it relies upon soil data rescue from a myriad of fragmented analogue soil data holdings worldwide to a globally coherent and complete soil information product.

Rescuing soil data includes three major steps: 1) the maintenance of libraries and holdings including scanning of thousands and thousands of analogue paper reports and maps into digital formats and assigning metadata to each object, allowing each object to be queryable, accessible and available online. In addition, it is also ensuring the safety of the data through proper backup of existing digital data entries. 2) compilation of the soil data under a common standard from the rescued data sources. This is done by entry and collation of legacy soil profiles and data (e.g. lineage, point location and year of recording, soil classification and, for soil depth intervals, soil morphologic observations and soil analytical measurements including values, units and methods used) from soil reports into a dedicated soil profile database and by digitizing legacy soil maps from published paper soil maps into a digital soil polygon database, followed by data standardization, harmonization and quality control.

3) when compiled under a common standard the legacy data are then used to generate gridded soil property maps within the GlobalSoilMap initiative according to the GlobalSoilMap specifications [%^11^]. The gridded maps are subsequently made freely available online to a wider user community. This community is potentially very large and includes soil scientists and soil mappers, agronomists, climate change modelers, biodiversity conservation specialists, economists, hydrologists, land-use planners, governments and policy makers, among others.

In this paper we provide an overview of the recent soil data rescuing activities linked to the GlobalSoilMap project and other international and national initiatives. Finally, we give some examples of success stories at the world, continental and country level from selected projects that achieved Soil Grids or final GlobalSoilMap products, thereby demonstrating the importance of data rescue activities of existing soil data.

## 2 Digital Soil Mapping, by GlobalSoilMap and other initiatives, and its use of soil profile point data

The GlobalSoilMap group was formed as an outgrowth of the International Union of Soil Sciences (IUSS) Working Group for Digital Soil Mapping with the purpose of providing consistently produced soil property information at 90m resolution across the world to aid in solving some of the key environment and societal issues including food security, global climate change, land degradation and carbon sequestration. The idea for the project was initiated at the 2006 IUSS Working Group for Digital Soil Mapping held in Rio de Janeiro, Brazil. A meeting of the working group to more formalize the concept was then held at the World Congress of Soil Science in Philadelphia shortly after the Rio meeting. In December of 2006, a meeting was called by key members of the soil science community at the Earth Institute at Columbia University to further discuss the concept. From these discussions, a foundational concept for how a global project could be structured was formulated. Over the next few years progress included signing a GlobalSoilMap consortium agreement, securing funding for producing data in Sub Saharan Africa, thanks to a grant from the Bill and Melinda Gates’ Fundation, and producing project standards and specifications. The first international conference on GlobalSoilMap was held in Orleans, France in 2013. In 2016, the IUSS established a GlobalSoilMap working group under the IUSS commission 1.5 ‘Pedometrics’.

Dissemination of soil profile data, at point locations, is in many countries strongly hampered by legislations concerning soil privacy and ownership, except for increasing numbers of countries and institutions which acknowledge the importance for sharing the data and results from publicly funded works (e.g., United States Department of Agriculture-Natural Resources Conservation Service (USDA-NRCS), ISRIC (International Soil Reference and Information Centre- World Soil Information, the European Soil Data Centre (ESDAC). A way to overcome this problem, which the project since the beginning aimed for, is to develop a globally distributed soil profiles database where the data are being managed by the data owners and made online and queryable through interoperable standards as defined by the community and in process of development. Another way is to compile and share the relatively still limited number of publicly available soil profile data and use those for global mapping. A third alternative is to only share and distribute the final soil data products, containing the predicted soil properties in a gridded format, without giving access to the original soil profile point data that was used for these predictions. The final GlobalSoilMap product represents an updateable outcome i.e. when new or additional soil profile data are available a new updated soil map can be quickly produced thus continuously improving the accuracy of the collaborative product.

The final product will be a globally and harmonized distributed grid map. However, besides data availability, achieving these global results would require distributed datasets to be harmonized at national, continental and global levels [e.g. 12–14]. In order to achieve this goal the GlobalSoilMap project developed guidelines and specifications [%^11^]. Distributed and strong computational capacities are needed to generate the maps at aimed for resolution.

Regardless of being national, continental and/or global, the following data rescue and grid map production steps are generally necessary, including references to GlobalSoilMap specific activities:

1 Identify and rescue legacy soil reports and maps and make digital scans with metadata publicly available (analogue carriers of data),
2a Capture and rescue legacy soil profile data from soil reports into digital soil point datasets, including geo-referencing,
2b Capture and rescue legacy soil maps into digital soil polygon datasets (i.e., build a vector dataset by vectorization of scanned (rasterized) data in a GIS),
3a Transform the original data in a common standard, for defining the soil property, the soil property measurement method and the units of expression,
3b Transform the standardized data from the original sequences of depth intervals to the standard sequence of soil depth intervals as defined by the GlobalSoilMap specifications,
4 Harmonize the data from the procedures and methods originally used to data according to reference procedures and methods conforming to the GlobalSoilMap specifications,
5 Assemble spatially exhaustive co-variates (e.g. from digital elevation models (DEM), remote sensing imagery, geological maps, vegetation maps; legacy soil type maps) including co-variates at a 3 arc-second resolution required for meeting GlobalSoilMap specifications,
6 Develop digital soil mapping models to predict soil properties, according to GlobalSoilMap specifications on a 3 arc-second grid.
7 Produce the maps including maps of the uncertainties,
8 Assess accuracy and validate the predicted soil property maps,
9 Deliver soil grid data products according to the GlobalSoilMap specifications.

A general framework has been proposed by Minasny and McBratney [%^15^] and the complete process is fully described in the GlobalSoilMap specifications [%^11^] and in a synthesis paper [7]. In this paper, we illustrate steps 1 to 4 and the efforts made for rescuing the primary soil data; we then provide a few examples of success stories achieving final products derived from the rescued primary data (steps 5-8) and we discuss the potential of future soil profile data rescue and the main issues related to their 9.use.

## 3 Synthesis of legacy soil profile data

Table 1 illustrates the progress in soil profile data rescue at various geographical levels from 2009 to 2015. This tremendous effort in soil profile data rescue resulted in nearly doubling the number of soil profiles stored in country databases. At the world level, (ISRIC- World Soil Information Service (WoSIS) database), the increase is tenfold [16-18; 134] and those data are, for the GlobalSoilMap properties, all standardized and available at www.isric.org/explore/wosis/accessing-wosis-derived-datasets. In absolute terms, the total of soil profiles existing and stored in the selected countries databases is obviously much higher and is currently about 800,000. Regrettably, large numbers of soil profiles stored in many country databases are yet not standardized and harmonized according to a global standard and are not shared. Note that the numbers given in the table of soil profiles at the world level, at the continental level (ISRIC [%^16^-%^18^], Sub-Saharan Africa [%^19^-%^21^], Latin America and Caribbean [%^22^], European Union [%^23^-%^26^]) and at the country level cannot be summed together. Large numbers of profiles compiled in the world database originate from the continental databases which originate to large extents from the national ones and from national survey reports. The difference in the number of data in the WoSIS database (World Soil Information Service) and the continental databases compared to the selected countries data is likely due to the time and capacity needed to identify the data sources and to capture, translate and harmonize the data, which is a job most efficiently and effectively done by the national data holders. Indeed, as stated by Rossiter [%^27^], much of the data are still proprietary and regrettably not generally accessible and unfortunately the question of open access to primary soil data is not resolved. Nevertheless, considerable successful efforts have been made since 2009 by ISRIC to rescue and add value to soil data in many countries where quality soil data have been generated and reported over the years, but where the data infrastructure is not up to standards and the data is in great danger of being lost (e.g. Sub-Saharan countries, [%^19^-%^21^]). Overall, we observe large discrepancies between countries, either in the total number of soil profiles compiled or in the efforts put in place in data rescuing, over the years 2009 and 2015 [%^28^-%^94^]. Table 2 provides the links to databases when they are available on the web. Database models and management systems are described by Batjes [17, 18, 134] at the world level, by Leenaars et al., 19,20] for Africa and by Hiederer [%^23^] and Hollis et al., [%^25^] for Europe.

Table 1

List of soil profile data rescue between 2009 and 2015 for selected countries and at world and continetal level

**Tab 1.1:** Global and continental datadanses

Geographical level	area in km^2^	Number of soil profiles in 2009	Number of soil profiles in 2015	number of new profiles	% of increase	key references
World					
World	130 000 000	10 250	117 446	107 196	1 046	[%^16^-%^18^]
Continental					
Sub-Saharian Africa	23 589 596	0	18 532	18 532	uncalculable	[%^19^-%^21^]
Latin America and carabean	20 199 984 sum of the 20 countries in SISLAC	unknown	6 099	unknown	uncalculable	[22]
European Union	4 500 000					
Europe (18 countries: Albania, Belgium, Denmark, Denmark, France, Greece, Hungary, Italy, Italy, Slovak Republic, Luxembourg, Netherlands, Portugal, Romania, United Kingdom, Slovenia, Spain, Switzerland)	3 000 000 (the extension of the participating countries)	560	560	0	0	[23]
Europe (18 countries: Albania, Belgium, Denmark, Denmark, France, Greece, Hungary, Italy, Italy, Slovak Republic, Luxembourg, Netherlands, Portugal, Romania, United Kingdom, Slovenia, Spain,Switzerland)	3 000 000 (the extension of the participating countries)	588	588	0	0	[24]
Europe (19 Countries: Belgium and Luxembourg, Denmark, England Wales Scotland, Finland, Germany, Italy, Netherlands, Portugal, France, Ireland, Bulgaria, Estonia, France, Hungary, Ireland, Romania, Slovakia and Switzerland)	3 000 000 (the extension of the participating countries)3	1 897	1 897	0	0	[25]
Europe (28 Countres: EU + Norway, Albania, Switzerland)	4 500 000 (whole EU plus Norway, Albania, Switzerland)	1 078	1 078	0	0	[26]

**Tab 1.2:** Countries databases

Geographical level	area in km^2^	Number of soil profiles in 2009	Number of soil profiles in 2015	number of new profiles	% of increase	key references
Argentina	2 780 400	0	2200	2200	0	
Australia	7 692 060	281 202	290 000	798	0	[28–29]
Belgium	30 528	7 020	7766	746	11	[30]
Cameroon	475 000	unknown	1040	unknown	uncalculable	
Chile	756 102	0	400	400		[31]
China	9 629 091	23 000	25 300	2300	10	[32]
Brazil	8 515 767	unknown	6 456	unknown	uncalculable	[33–36]
Canada	9 984 670	4 050	8 615	4 565	113	[37–39]
Mexico	1 964 375	22 430	22 430	0	0	[40]
France (mainland)	551 500	37 937	64 123	26 186	69	[41–42]
France (French west Indies)	2 835	148	682	554	374	[43]
France (La Réunion)	2 512	0	256	256	uncalculable	[43]
France (Guyana)	91 000	0	256	256	uncalculable	[43]
Slovakia	49 035	1 871	18 171	0	0	[92]
Denmark (Greenland)	2 166 086	0	650	650	uncalculable	
(Denmark (mainland)	43 094	2 250	12 456	10 206	454	[44–45]
Croatia	56 594	6 500	6 500	0	0	
Russia	17 098 242	0	863	863	uncalculable	[46]
Indonesia	1 910 931	0	30 867	30 867	uncalculable	[47]
Portugal	92 090	0	3 470	3 470	uncalculable	[48]
Scotland	77 800	14 722	14 722	0	0	[93–94]
Thailand	513 120	244	300	66	27	
USA	9 629 091	37 937	64 123	26 186	69	[49–55]
South Korea	99 828	390	405	15	4	[56–58]
The Netherlands	37 354	7 859	7 965	106	1	[59–60]
Hungary	93 030	10 898	45 068	34 170	314	[61–64]
Ireland	70 273	430	667	237	55	[65–66]
Finland	338 424	36	36	0	0	[67]
Iran	1 648 195	0	25 909	25 909	uncalculable	[68]
Japan	377 930	0	7 150	7 150	uncalculable	[69]
India	3 287 363	88 900	91 900	3 000	3	
Nigeria	923 768	1 634	1 825	191	12	[70–73]
England&Wales	151 000	5 518	10 796	5 278	96	[74–75]
New Zealand	270 467	2 990	7 651	4 661	156	[76–79]
Greece	131 957	0	200	200	uncalculable	[80]
Romania	238 391	3 338	3 839	501	15	[81–84]
Switzerland	41 290	0	6 000	6 000	uncalculable	[92]
Ukraine	603 548	1 500	2 075	575	38	[85]
Uruguay	176 215	1 386	1 556	170	12	
NorthenTunisia	2 822	0	180	180	uncalculable	[86]
Latvia	64 589	0	746	746	uncalculable	[87]
Luxembourg	2 593	805	860	55	7	
Morocco	710 850	394	1 106	712	181	
Sri Lanka	65 610	118	118	0	0	[88–90]
Slovenia	20 273	1 899	1 975	76	4	
Czech Republic	78 866	3 500	4 110	610	17	[84]
South Africa	1 220 000	16 000	17 750	1 750	11	[91]
Total world databases			10 250	117 456	107 206	1046
Total countries data bases			565 507	821 533	256 026	45	

**Tab 2:** Links to national databases available on the web

Geographical level	name of the database	web site
World	WoSIS (World Soil Information Service)	http://www.isric.org/data/wosis
World	ISRIC-WISE Global Soil Profile Data	http://www.isric.org/data/isric-wise-derived-soil-property-estimates-30--s 30-arcsec-global-grid-wise30sec
Continental		
Sub-Saharian Africa Latin America and carabean	AfSP (Africa Soil Profiles database) SISLAC	http://www.isric.org/data/africa-soil-profiles-database-version-01-2www.sislac.org
European Union		
Europe (18 countries: Albania, Belgium, Denmark, Denmark, France, Greece, Hungary, Italy, Italy, Slovak Republic, Luxembourg, Netherlands, Portugal, Romania, United Kingdom, Slovenia, Spain, Switzerland)	SPADE/M : Soil Profile Analytical Database of Europe of Measured parameters	http://esdac.jrc.ec.europa.eu/content/spadem
Europe (18 countries: Albania, Belgium, Denmark, Denmark, France, Greece, Hungary, Italy, Italy, Slovak Republic, Luxembourg, Netherlands, Portugal, Romania, United Kingdom, Slovenia, Spain,Switzerland)	SPADE-1: Soil Profiles in Europe	http://esdac.jrc.ec.europa.eu/content/european-soil-database-v20-vector-and-attribute-data
Europe (19 Countries: Belgium and Luxembourg, Denmark, England Wales Scotland, Finland, Germany, Italy, Netherlands, Portugal, France, Ireland, Bulgaria, Estonia, France, Hungary, Ireland, Romania, Slovakia and Switzerland)	SPADE-2: Soil Profiles in Europe	http://esdac.jrc.ec.europa.eu/content/soil-profile-analytical-database-2
Europe (28 Countres: EU + Norway, Albania,Switzerland)	SPADE-14: SOIL PROFILE ANALYTICAL DATABASE	Not yet available
Countries		
Argentina	Sistema de Informacion de Suelos de INTA	http://sisinta.inta.gob.ar/
Australia	National soil site data collation (NSSDC)	http://www.clw.csiro.au/aclep/soilandlandscapegrid/index.html
Belgium	Databank Ondergrond Vlaanderen (DOV)	dov.vlaanderen.be
Cameroon	Ongoing Digital Soil mapping Project for Cameroon (University of Dschang and IITA Cameroon)	Not kown yet
Chile		
China	China Soil Database	http://vdb3.soil.csdb.cn/
Brazil	Sistema de Informagao de Solos Brasileiros & ESALQ Brazilian Soil Profile Database	https://www.bdsolos.cnptia.embrapa.br/consulta_publica.html & http://www.esalq.usp.br/gerd
Canada	Canadian Soil Information Service	http://sis.agr.gc.ca/cansis/
	Canadian Digital Soil Data Consortium	http://soilinfo.ca/
Mexico	Natinal Forest Inventory formacion Nacional sobre Perfiles de Suelo (Serie I)	http : //www.inegi.org.mx/geo/contenidos/recnat/edafologia/vectorial_seriei.aspx
	Conjunto de Datos de Perfiles de Suelos Escala 1: 250 000 Serie II (Continuo Nacional)	http://www.inegi.org.mx/geo/contenidos/recnat/edafologia/vectorial_serieii.aspx
France (mainland)	soil profiles in the 1:50,000 maps database DoneSol	www.gissol.fr
France (French west Indies)	Donesol and Valsol	www.gissol.fr
Geographical level	name of the database	web site
France (La Réunion)	Donesol and Valsol	www.gissol.fr
France (Guyana)	Donesol and Valsol	www.gissol.fr
France (New-caledonia)	Valsol	www.gissol.fr
Slovakia	National Agricultural Soils Inventory Database (AISOP), agricultural soil dadatabe, foest soil datadase	
Denmark (Greenland)		
Denmark (mainland)	Danish Soil Profile Database	
	Wetland database	SINKS
Croatia	National Soil Database of Croatia	no website
Russia	Unique State Registr of Soil Resources of Russia	http://atlas.mcx.ru/materials/egrpr/content/1DB.html
Indonesia	SIMADAS (Sistem Informasi Manajemen Data Sumberdaya Lahan)	
Portugal	INFOSOLO	
Scotland	Scottish Soil Database	http://www.soils-scotland.gov.uk/data/nsis
Thailand	Thailand soil database	www.ldd.go.th
USA	NCSS Microsoft Access Soil Characterization Database	http://ncsslabdatamart.sc.egov.usda.gov/
South Korea	Korean Soil Database	http://soil.rda.go.kr
The Netherlands	BIS Nederland	www.bodemdata.nl
Hungary	Digital Kreybig Soil Information System (DKSIS)	http://medaphon.rissac.hu/kreybig/login/login_ui.php; http://maps.rissac.hu/kreybig_bodrogkoz/
	MARTHA ( Hungarian Detailed Soil Physical and Hydrological Database)	https://www.researchgate.net/publication/250979646_Introduction_of_the_Hungarian_Detailed_Soil_Hydrophysical_Database_MARTHA_and_its_use_to_test_external_pedotransfer_functions
	TIM - talajinformációs és monitoring rendszer - Soil information and monitoring network	http://portal.nebih.gov.hu/-/a-tim-azaz-a-talajvedelmi-informacios-esmonitoring-rendszer-
Ireland	Irish Soil Information System	www.http://erc.epa.ie/safer/
Finland	Finnish Soildatabase 1:250 000	http://www.paikkatietohakemisto.fi/geonetwork/srv/fi/main.home
Iran	INSDB=Iran National soil Data Base	http://www.insdb.swri.ir
Japan	Soil Information Web viewer	http://agrimesh.dc.affrc.go.jp/soil_db/
India	Bhoomi (tentative name)	http://www.nbsslup.in/ (under construction)
Nigeria	Nigeria Soil Dbase	
England&Wales	LandIS - Land Information System (for England and Wales)	www.landis.org.uk
New Zealand	National Soil Data Repository (NSDR)	https://soils.landcareresearch.co.nz/
Greece	elgo soil data base	www.gssoil-nagref.gr
Romania	PROFISOL	
Switzerland	Soil Information System NABODAT	www.nabodat.ch
Romania	MoniSol-RO	
Ukraine	Ukraine Soil Properties Database	
Uruguay		
NorthenTunisia		
Latvia	Digital Land and Soil Database of Latvia	Not known yet
Luxembourg	BD_SOL	Not known yet
Morocco	Moroccan Soil Profile Database	
Sri Lanka	SICANSOL	No known yet
Slovenia	Several databases and data collections available at three institutions.	http://www.kis.si/eTLA
Czech Republic	PUGIS	http://pedologie.czu.cz/
South Africa	South African Soil Profile Database	www.arc.agric.za

**Figure 1:**
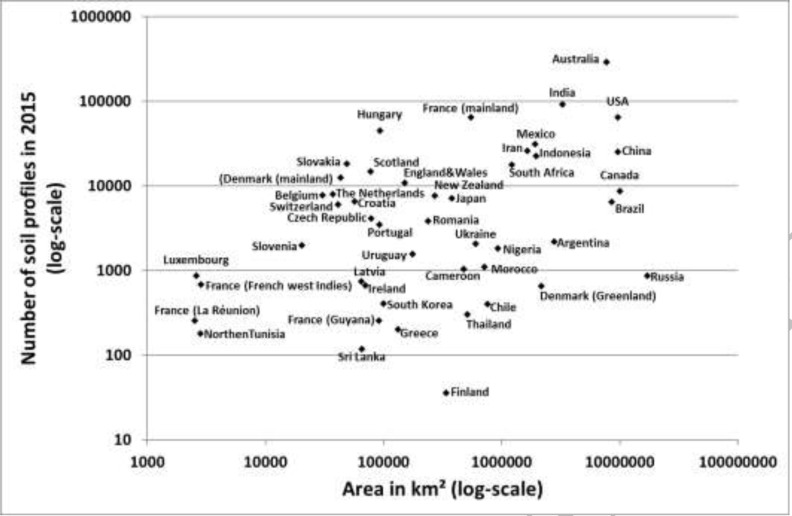
Log-Log scatterplot of countries areas versus soil profiles

Figure 1 shows the relation between the total surface area of the selected countries and i) the total number of soil profiles stored in their database and ii) the soil profiles rescued between 2009 and 2015. As expected, there is no clear relation between a country’ area and data rescuing effort. Some rather small countries are in a very advanced stage of data rescuing (e.g., Belgium [30], The Netherlands [59–60], Denmark [44–45]), whereas some very large countries are just beginning their data rescuing efforts (e.g., Russia [46]).

## 4 Soil profile data rescue efforts

In the following sections, we present a few of many soil profile data rescue efforts. We focus on data rescue efforts that have led to final products in line with the GlobalSoilMap specifications.

### 4.1 Case studies at the world level

#### 4.1.1 WoSIS data (World Soil Information Service)

The World Soil Information Service (WoSIS) database is developed at ISRIC [134] within the conceptual framework of the Global Soil Information Facility which facilitates collaborative bottom-up initiatives to process and exchange soil data at the global level (www.isric.org/explore/wosis). Ideally, primary soil profile data are being managed and maintained by the national data owners whereby the data are connected and made queryable online by an interoperable infrastructure through data exchange standards. Since 2009 these standards continue to be defined and developed by the global soil community, but is a very slow process. Anticipating these standards being developed further, the configuration of WoSIS is that of a centralized database which accommodates current, more conventional, data exchange mechanisms between collaborative organizations to collate and harmonize soil data and which therewith meets both short term and long term goals of collaborative soil mapping.

**Figure 2:**
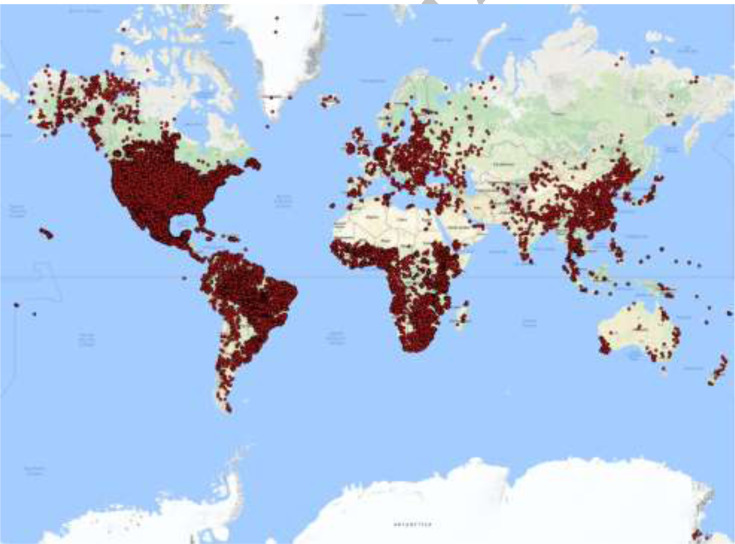
Location of the soil profiles rescued in WoSIS

The databases at the higher level (world, continent) are actually compilations of data, under a common standard, from databases and reports originating at the lower level (national and subnational) shared by collaborative partner organizations. So far, one snapshot of the WoSIS data has been released in July 2016 (http://geonode.isric.org/layers/geonode:wosis 201607 profiles). The world level data are spatially irregularly distributed, with some parts of the world being relatively dense while other parts having still very sparse point data or no data at all (Fig. 2).

This distribution is strongly related to the amount of data previously shared through collaborative projects and to the amounts of data currently published by the various countries and institutions due to current and recent data policies, but is also influenced by limited capacities and a prioritization of the effort. Very large differences are observed between densities at the country level and the density at the world level (for instance in France, Iran, Indonesia). More generally, we hope that a map such as presented in Figure 2 will encourage countries to collaborate through a bottom-up approach and to provide data access to WoSIS and/or to develop and share their own country level products according to the GlobalSoilMap specifications similar to the most recent ones developed in some countries [e.g. France, Scotland, USA, Australia, Denmark]. The WoSIS data collection effort has proven to be very useful in producing the first world-wide SoilGrids at 1 km resolution [16] followed by a world-wide grid at a 250 m resolution [95]. These global grids were preceded by grids at similar resolution for the Sub- Saharan Africa region [96–97] using the data compiled in the African Soil Profiles database [19–21].

#### 4.1.2 SoilGrid-250m

A new worldwide SoilGrids-250m has just been released ([95];http://www.soilgrids.org). The new version of SoilGrids predictions comes with an open data license. SoilGrids data are available for viewing and download via the data portal at http://www.soilgrids.org and can also be accessed through web coverage services. A bottom-up approach has been applied to rescue and use the soil profile data available from the country level and a top-down approach for producing the gridded maps through global modelling. A fully bottom-up approach (i.e., both data rescuing and subsequent modelling are done at country level) including the rescue and use of the large amounts of not yet publicly accessible soil profile data available at country level. A few initiatives have been initiated to encourage in-country capacity building for data rescue and subsequent digital soil mapping process. Top-down approaches will still be used within the collaborative global consortium to fill gaps where bottom-up approaches are not yet feasible. The global SoilGrids-250m would also serve as covariate and help harmonization between country level products and development of ensemble methods, mixing different predictions (e.g. [98]).

### 4.2 Case studies at the continental level

#### 4.2.1 Europe

In Europe, several soil profile databases have been developed, covering countries belonging to the EU and other bordering countries, for example, SPADE2 (http://esdac.jrc.ec.europa.eu/content/soil-profile-analytical-database-2). This database includes around 1,800 soil profiles covering the following countries: Belgium and Luxembourg, Denmark, England and Wales, Finland, Germany, Italy, Netherlands, Portugal and Scotland [23–26].

LUCAS is a topsoil database at European scale including more than 22,000 soil samples from the 27 member states of the European Union [99–103]. In 2009, the European Commission extended the periodic Land Use/Land Cover Area Frame Survey (LUCAS) to sample the main properties of topsoil (0-30 cm) in 25 Member States of the European Union. This sampling exercise has been extended in Romania and Bulgaria in 2012. The samples have been analyzed and the compiled LUCAS-topsoil database is available in European Soil Data Centre (ESDAC). The LUCAS soil sampling campaign was repeated in 2015 and the data will become available in 2017.

#### 4.2.3 Sub-Saharan Africa

**Figure 3:**
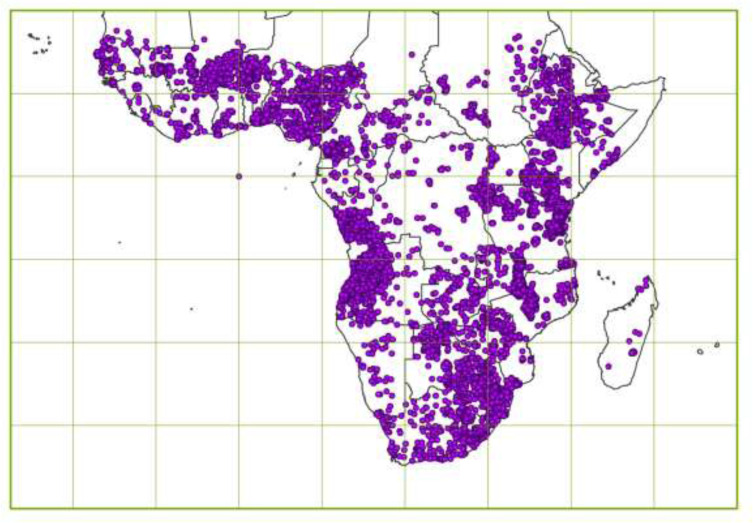
Location of the data rescued in the Sub-Saharan Africa Soil Profiles database

The Africa Soil Profiles database [19–21], version 1.2, compiles standardized and original soil data from 18,572 soil profiles of Sub-Saharan Africa, of which 17,160 are georeferenced (Fig. 3).

The data were captured from 540 data sources with full lineage specified; about 25% of the profiles were extracted from earlier ISRIC datasets, 30% from other digital datasets and 45% from analogue reports (503). It includes data for approximately 140 soil properties, including soil analytical data measured in over 100 specified laboratories, using over 350 specified laboratory methods. The original values were standardized according to standard data conventions (Soil and Terrain database, SOTER, http://www.isric.org/projects/soil-and-terrain-database-soter-programme) for 25 soil properties observed from the profile and the profile site and for 75 soil properties, both morphologically observed and analytically measured, reported from the soil profile layer features (depth intervals; 4 on average to 110 cm depth on average). The standardized values for some 60 soil analytical properties, evaluated in the laboratory, were subjected to routine quality assurance protocols. The temporal distribution of the data spans over 60 years peaking in the 1980’s, and the spatial distribution of the data covers 40 countries. The Africa Soil Profiles database [19–21] is compiled within the context of the Africa Soil Information Service (AfSIS) project, with collaborative contributions from Cameroon, Nigeria, Ghana, Mali, Ethiopia, Kenya, Tanzania and Malawi, and is accessible at www.isric.org/content/africa-soil-profiles-database and http://africasoils.net/services/data/soil-databases/africa-soil-profile-database/). At present, the effort is ongoing through collaboration with bottom-up initiatives of organizations in a number of SSA countries (i.e., Ghana, Cameroon, Burkina Faso).

The data rescue in Sub-Saharan Africa has resulted in gridded soil maps for all primary and derived soil properties mentioned in the GlobalSoilMap specifications [11], including electrical conductivity, bulk density, plant-available water holding capacity and depth l to bedrock and effective root zone depth (for maize) [104–107]; In this region, legacy data proved particularly relevant, compared to newly sampled topsoil data, 1) to allow cost effective mapping detailed and consistent at both the continental and national extent and 2) to assess the effective depth and volume of the soil in which soil water and nutrients are retained and in which plants do actually grow. These Africa SoilGrids were used as input for yield gap analyses and quantitative evaluation of the fertility of soils.

### 4.3 Case studies at the national level

#### 4.3.1 United States of America

**Figure 4:**
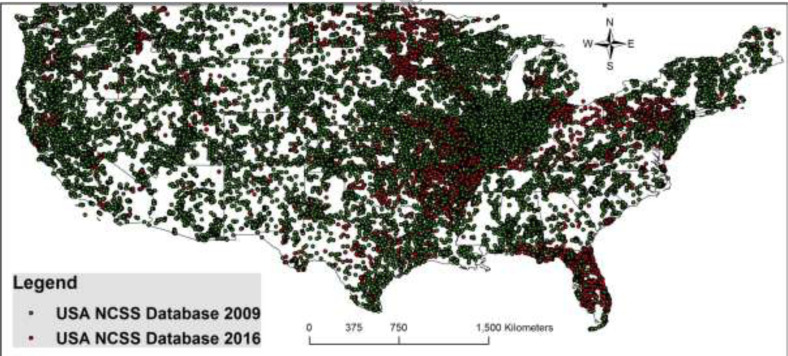
USA National Cooperative Soil Survey soil profile data rescued between 2009 and 2016. Green dots represent the 2009 soil profile data and the red dots represent the 2016 soil profile data showing an increase in their number from 29, 130 to 60, 962.

In the United States of America (USA), a tremendous effort led to an approximate doubling of the number of soil profiles between 2000 to 2016 (Fig. 4). The majority of the rescued data came from Universities that collected and analyzed the data during the field soil survey campaigns under cooperative agreements with the USA national Cooperative Soil Survey [108]. Some historical data were also rescued [109]

#### 4.3.2 France

**Figure 5:**
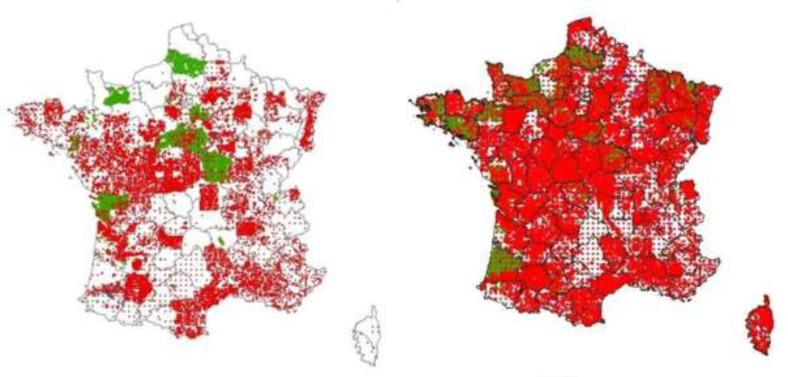
Rescued soil profiles in France between 2009 (left) and 2015 (right) (France). Complete soil profiles with full description are in red, auger borings are in green. The total number of points in 2009 is 76, 400, and 160, 103 in 2015.

In France, an important data rescue effort led to a 69% increase of the number of soil profiles data from 2009 to 2015 (Fig. 5) [41–42] giving an impressive coverage at adequate density of the French territory.

#### 4.2.2 Australia

**Figure 6:**
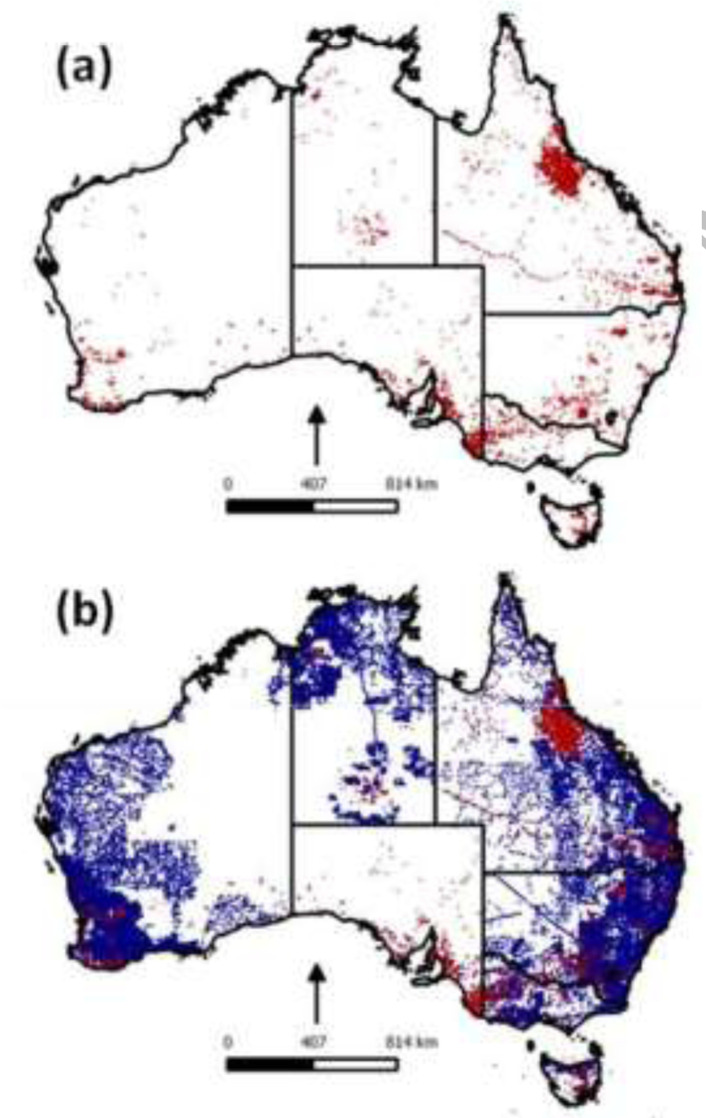
(a) Distribution of sites contained in the previously existing national NatSoil Database of Australia (11, 500 sites) and (b) distribution of sites contained in the new National Site Data Collation (NSSC) database (281, 000 Sites)

Australia has a rich but non-uniform and incomplete archive of existing soil mapping and site data. The state and territory government agencies are primarily responsible for the collection and management of soil data within their territories, in addition, CSIRO, Universities and Geoscience institutions have collected data and hold records. Thus, there are at least 13 independent and unique soils data management systems, some eight with formal responsibilities for regional, national or specific data [28]. For at least the last 70 years, these agencies have been collecting soil site data, and for some 40 years have used various forms of data systems (in most cases developed within the institution). Before the GlobalSoilMap project initiation, these soil site datasets were not compiled into a consistent data set conforming to a single standard. The GlobalSoilMap project provided the impetus for combining some 281,000 soil profiles into a single uniform database using data interoperability approaches and a consistent database schema for the project data collation [28–29]. Also contained in this database are 2.5 million laboratory measurements. Figure 6 shows the progress between 2009 (the launch of the GlobalSoilMap project) and 2015. Very large areas that had very sparse information in a consistent national collation (for instance in western and northern parts of the country) are now covered by a large amount of soil profile data now available for new mapping and estimation

#### 4.3.3 Other

It was found that some countries not only rescue soil profile data but also soil descriptions captured by hand auger borings. This is partly the case for France (see Fig. 5). The Netherlands is and outstanding example where more than 327,000 auger descriptions have been rescued, leading to a total density of observation points of about 13 km^-2^ in agricultural, forest and natural lands. These auger descriptions are very supportive in predicting the spatial distribution of soil types and soil properties. For instance, a recent use of this data led to probability mapping of iron pan presence in sandy podzols in South-West France [135].

## 5 Soil map data rescue efforts

Legacy soil maps are available in quite a large number of countries and are a valuable soil covariate along with soil profile point data, for use in digital soil mapping. Therefore, soil maps from legacy soil survey data holdings across the world are being rescued and compiled and serve as input for a number of countries to developing techniques for digital soil mapping. This legacy information contributes through a bottom-up approach to a common, consistent and geographically contiguous applicable dataset of relevant soil properties covering the planet’s land surface. The legacy soil data holdings, including tens of thousands of published soil maps and associated reports, have been produced over an extended period of time by numerous institutions using different methods, standards, scales and taxonomic classification systems.

### 5.1 Case studies at the world and continental level

The largest collection of soil survey archives publicly accessible online is the ISRIC - World Soil Information document database (library: http://www.isric.org/content/search-library-and-map-collection). The ISRIC library has built up a collection of nearly 35,000 maps, reports and books. The many soil maps accompanied by the associated soil reports and related thematic information provide rich soil survey data and complementary information. Much of these materials, each with a unique identifier and full metadata, has been scanned through a huge effort since 2009, including an effort at the EU level. This resulted in the Digital Archive of Soil Maps (EuDASM) which includes around 6,000 maps from the ISRIC library for 140 countries worldwide [110], and can be queried and accessed online at the ISRIC website. EuDASM is available in the European Soil Data Centre (ESDAC)at:(http://esdac.jrc.ec.europa.eu/resource-type/national-soil-maps-eudasm).

The Food and Agriculture Organization (FAO) has recently finished uploading 1228 soil and land legacy maps (mainly soil maps, but also land use, geological and land cover legacy maps): http://www.fao.org/soils-portal/soil-survey/soil-maps-and-databases/fao-soil-legacy-maps/en/.

**Figure 7:**
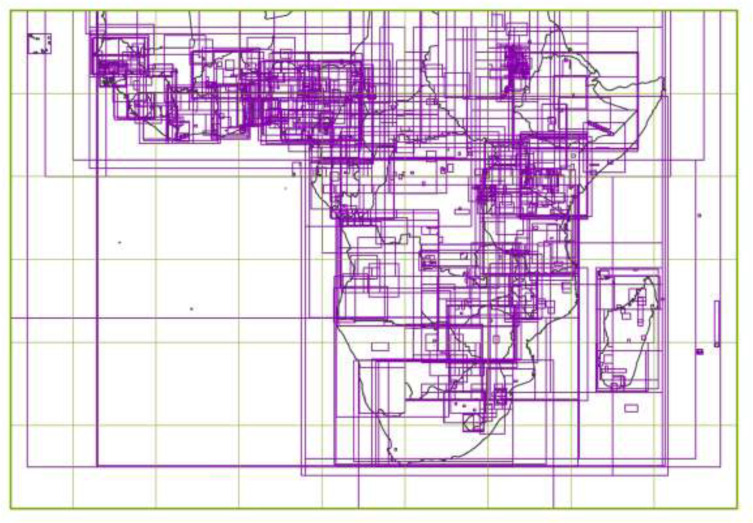
Contour map of the (Sub-Saharan) Africa Soil Maps database.

During the AfSIS/GlobalSoilMap project [19–21], thousands of selected soil reports and maps of Sub- Saharan Africa were scanned at ISRIC and made available online. Moreover, thousands of additional soil maps, and associated soil reports, of Africa were identified from other libraries and holdings in Europe and Africa (i.e., IRD, WOSSAC, FAO, UGhent) and after duplicate removal were added to the ISRIC library collection, including online access to digital scans with full metadata (Fig. 7).

The Africa Soil Maps database represents a spatial inventory of approximately 5,000 legacy soil maps recently made available online at the ISRIC library. Soil maps originating from six European archives and a few African national countries were identified and added to the library through a large effort to harmonize metadata and exclude duplicates (Figure 7). Some legacy soil maps that had been scanned have also been digitized into a GIS-database format, including information about the topology, geometry and legends. The Malawi data has been used by ISRIC for producing a Soil and Terrain (SoTer) database [111].

### 5.2 Case studies at the national level

#### 5.2.1 Nigeria

For Nigeria, soil data holdings have been identified and collected from various libraries, including numerous analogue soil reports and maps from the ISRIC library, a digital soil GIS-map from the University of Amsterdam and a few items from holdings in Nigeria (Zaria University, Niger River Basin Management authority, Federal Department of Agricultural Land Resources). Selected items not yet in the ISRIC library were photocopied and brought to the Netherlands and added to the ISRIC collection, scanned (rescued) and brought online. For the AfSIS project, ISRIC digitized, georeferenced and compiled the soil data of 1,250 profiles from Nigeria into the Africa Soil Profiles database version 1.0 , [19–21], of which 45 profiles were available through earlier ISRIC databases (27 in ISIS and 19 in WISE). Georeferencing and data quality control proved to be major challenges in collating these legacy soil data, and are described in [70–71] the first soil mapping applications in [72]. The national database of Nigerian soil profiles currently contains about1,900 profiles, nearly 50% more soil profiles has been added since 2011 and used for a range of applications [72–74] and we expect these additional soil profile data from Nigeria to be made publicly available online with the original collaborative initiative.

#### 5.2.2 India

In India, the National Bureau of Soil Survey and Land Use Planning (NBSS&LUP), under the Indian Council of Agricultural Research (ICAR), is the agency for collecting and generating soil data in India. With a network of centers throughout the country, the agency has generated soil resources maps at the 1:1,000,000 scale at the country level, at the 1:250,000 at state and union territory levels, at 1:50,000 for 83 out of 640 districts, and at 1:5,000 scale for 70 watersheds. These resource maps provide layer-wise soil information on soil texture, organic carbon contents, pH, nutrients, cation exchange capacity and in limited cases, water holding capacity. There are few other organizations who also compile such data; however, a harmonized and searchable soil database is yet to be developed.

#### 5.2.3 Indonesia

In Indonesia, soil resource inventories have been conducted since 1905 by the Indonesian Centre for Agricultural Land Resource Research and Development (ICALRD) and its colonial and post-independence predecessors for various purposes (e.g. agricultural planning, erosion hazard assessment, and soil fertility monitoring). This has resulted in soil survey reports and soil maps (e.g. [47]). Various databases have been developed to store soil data in Indonesia. As of 2016, 100% of Indonesia is covered by a 1:250,000 scale map and 40% by detailed maps (≤1:50,000 scale). In addition, a land system map at the scale of 1:250,000 is available for the whole country and there is an ongoing effort to scan soil survey reports and hardcopy maps.

#### 5.2.4 South Korea

In South Korea, detailed soil maps (1:25,000) are now available for the entire country, both in hard copies and digital format. Furthermore, highly detailed soil maps (1:5,000), surveyed from 1995 to 1999 for the entire country, were digitized and made available for the public, through the website (http://soil.rda.go.kr). Two soil databases were constructed, as part of the soil information system of Korea. The first is a spatial database of computerized soil maps at a variety of scales (1:250,000, 1:50,000, 1:25,000, and 1:5,000). The second database is a parcel-based soil fertility (chemical properties) database, containing around 7,000,000 data objects.

#### 5.2.5 United States of America

In the USA, a “Digital Collection of Selected Historical Publications on Soil Survey and Soil Classification in the United States of America” was assembled comprising a selection of scanned maps, photographs, unpublished reports and government publications that provide some historical perspective on soil survey activities and the development of soil classification in the United States [109]. The scanned documents cover various topics such as tropical soils; the history of the National Cooperative Soil Survey; historical development and theory of soil classification; field excursions organized for 1st and 7th International Congresses of Soil Science; soil survey investigations; and Soil Taxonomy. The series of historical soil maps, 1909-1998, illustrates several conceptual changes in soil geography and soil classification at the national and regional (province-based) scales (http://www.nrcs.usda.gov/wps/portal/nrcs/detail/soils/survey/publication/). Also a large number of published soil survey manuscripts in paper format have been scanned and digitized and made publicly available at http://www.nrcs.usda.gov/wps/portal/nrcs/soilsurvey/soils/survey/state/ (accessed on August 27, 2016). Efforts to rescue documentations collected during soil survey campaigns (a field notes, pedon descriptions, transect data) are also underway and conducted at regional levels. For example, the project in Region 10 comprising of 8 states located in northcentral US has rescued and georeferenced close to 47,364 pedon descriptions [98] that are available on an ArcGIS platform (http://www.arcgis.com/home/webmap/viewer.html?webmap=80c4349331754aada7572c54a1377d66 &extent=-116.5399,36.0679,-84.0863,52.1478, accessed on July 27, 2016).

#### 5.2.6 France

In France, a preliminary analysis of national soil information and potential for delivering GlobalSoilMap products has been made in 2013 and published in 2014 [112]. At the end of 2015, a catalogue of 5,854 soil maps became available at http://www.gissol.fr/outils/refersols-340. About half of the collection is currently being digitized and 407 soil maps are accessible as complete database. This effort is a long-term ongoing process, with major emphasis on building a harmonized database. Priority is given to maps with scales ranging between 1:250,000 - 1:50,000. [41–42].

#### 5.2.7 Scotland

In Scotland, the 1:25,000 scale soil maps were created by the Macaulay Institute for Soil Research (now the James Hutton Institute) and are based on data collected mainly between 1947 and 1987. The soil classification has evolved since the 1940s and the updated maps follow the 2013 revised soil classification system. The 1:25,000 scale soil maps were created by the Macaulay Institute for Soil Research and are also based on data collected mainly between 1947 and 1987. Scotland has a major programme to update their 1:25,000 scale soil maps and make them available for download, see http://www.soils-scotland.gov.uk/data/soil-survey25k.php. Further information on how the maps were made, how the soils were classified and the state of progress of soil maps rescue can be found at http://www.soils-scotland.gov.uk/.

#### 5.2.8 Latvia

In Latvia, analogous soil maps (1976-1997) of agricultural land at the scale of 1:10, 000 were digitized and a database was created. The database consists of two data sets: 1) polygon characterization, including the year of mapping, soil type according to genetic classification and the textural group) and 2) soil profile data, including the year of mapping, soil type according to genetic classification, the textural group (topsoil, bottom layer), and integrated textural group (topsoil and bottom layer), pH value, depth of CaCO3. Altogether, the database contains data from 543601 polygons and 746 soil profile descriptions [87]. Some attempt was done to convert the soil units from National classification to the WRB 2014. The technical work is finished but the database is not yet publically available due to the discussions in which portal to place it and who will be responsible for its maintenance.

#### 5.2.9 Russia

In Russia, detailed soil maps, at scales 1:10, 000-1:50, 000, are available for all arable lands, both in hard and scanned copies. The total number of maps is about 20, 000. The majority of the maps is accompanied by explanatory notes with characteristics of main soils, and representative profiles description. The map collection is stored in the Soil Data Center of V.V. Dokuchaev Soil Science Institute (Moscow) and are not publicly available. They are used as an additional source for the development of the Unique State Register of Soils of Russia, and for different databases compilation. Additionally the Soil Data Center contains regional soil maps at scale 1:200,000 - 1:500,000 for the most regions of Russia, as well as near 140 sheets of State Soil Map of USSR (scale 1:1,000,000) with explanatory notes. Some of these maps were digitized, or updated based on digital soil mapping approaches [136].

#### 5.2.10 Hungary

Soil mapping has a long tradition in Hungary, several small scale soil maps were compiled in the first decades of the 20th century. Large scale mapping at a scale of 1:25, 000 started in the 1930s and continued till the end of the 1950s. Large scale mapping campaign at 1:10, 000 scale supporting the intensive large scale agriculture continued till the early 1980s. These datasets have been used as a source for smaller scale soil maps between 1:100, 000 and 1:1, 000, 000 scales. The 1:25, 000 scale maps have already been digitized, all the polygons and the related points has been organized into digital soil datasets. The 1:10, 000 scale maps are partially digitized, the process is still ongoing. Due to the tremendous amount of emerging soil profile data and new observations and to the innovative digital soil mapping tools being available, several new data products have been or being produced as new, independent data sources serving the new kind of data needs, and increasing the data diversity.

### 5.3 Usefulness and limitations of rescued soil maps for GlobalSoilMap

Soil properties can be derived from both detailed soil maps (generally a cartographic scale of 1:100,000 or more detailed) and soil point data (i.e. measurements down the soil profile at a georeferenced location). When using soil maps only, the most used methods are: extracting soil properties from a soil map, using a spatially weighted measure of central tendency (e.g. the mean), or spatial disaggregation of soil maps (e.g., [38, 54, 113–115]).

When only soil maps are available, soil properties can be extracted from soil maps according to the distributional concepts underlying the soil mapping units. In some cases, it will be appropriate to estimate soil properties using an area-weighted mean, as was done for example in the United States [51–52]. However, in most circumstances, the original soil map will have information on the factors controlling soil distribution within an individual map unit. This is most commonly based on terrain (e.g. a catena or characteristic toposequence). The widespread availability of fine-resolution terrain variables, now allows the soil properties to be ‘disaggregated’ at soil type levels occurring within soil mapping polygons. Recent examples of this kind of approach canbe found in [38, 54, 113–115].

An extension of this approach is to use areas where there is a detailed understanding of soil distribution as a basis for extrapolation to a broader domain, examples can be found in [116–118]. Moreover, soil map units and soil point data can be used together to improve gridded predictions of soil properties. Soil map units can be used as a co-variate for scorpan kriging (i.e. a prediction method using both spatial co-variates linked to the controlling factors of soil distribution and to the points location, [9]), for instance [119–124]. This often implies merging different soil map units in order to reduce their number [123–124]. Specific information can be extracted from soil maps (e.g., parent material, broad soil classes, soil textural classes, eg., [124]) and also used as a co-variate. This will often require some merging of classes too. Note that depending on the target soil property the most efficient merging of classes can differ and often requires the soil surveyor expert knowledge. For instance, in France, different parent material classifications may be used as co-variates for soil texture and for pH mapping [124]. Finally, independent predictions from soil maps and from point data can be merged and weighted through ensemble methods (e.g. [98]).

Using soil maps over large territories often requires huge harmonizing efforts. Indeed different soil maps may have been produced by different soil surveyors, having different objectives and various pedological concepts. The scales may also differ between soil maps. For instance huge efforts have been invested in harmonizing the European geographical Soil Database (e.g., [125]) and the US soil map (e.g., [108]). Attempts to update the world soil map using SOTER methodology are still ongoing in various parts of the world (e.g., [111; 126]).

Finally, even if soil maps cannot be considered as truly independent validation data, they are often useful to evaluate some gridded products and to check inconstancies between gridded predictions and expert delineations of broad soil classes

## 6 Success stories

The final goal of the project is to provide a global freely available high-resolution dataset on key soil properties which is either downloadable or accessible through web-services. This dataset will include 18 billion of point data on a 3x3-arcsec grid and 18 billion of block data on 3x3-arcsec cells (i.e., we predict soil properties and their uncertainties at each node of a 3x3-arcsec grid and their mean values and their uncertainties on 3x3-arcsec cells centered on the grid nodes), on six standard depths for 12 soil properties with associated uncertainties (90 % confidence interval). The project includes tiered specifications depending on the spatial entity (point or block) and on uncertainty and validation specifications [11].

### 6.1 World-level

SoilGrids (e.g., [16; 95]) are the first globally consistent and contiguous complete gridded soil properties maps of the world, derived from rescued legacy soil profile data through DSM techniques, and was released by ISRIC. Despite some limitations (grid cell area, and rather low accuracy in some areas); they constitute a first proof of concept and example on what can potentially be achieved at the world level. However, they do not describe sufficient variability at short distances. Despite these limitations at the local level, the SoilGrids provide key support for global modeling efforts.

Soilgrids250 m [95] was recently released on the ISRIC website, showing significant improvements compared to the 1 km product. ISRIC is waiting from feedback from countries. However, the number of soil profiles available for model calibration remained limited (only just over 100,000). One of the main advantages of releasing such products may be to identify the parts of the world where data is obviously missing. This may convince countries either to provide data to ISRIC and therewith to the global soil science community, to develop their own bottom-up products through collaborative efforts to fill the gaps, to correct the obvious errors or to simply enhance the accuracy where insufficient for national purposes. Obviously there will also be parts of the world where there will be no data at all or where data has been lost. SoilGrids will therefore be useful to fill these gaps. Another possibility is to collaborate by evaluating and validating global SoilGrids products with national profile datasets or predictions or to make national datasets available to improve the global predictions.

### 6.2 Continent-level

The situation in Sub-Saharan Africa is similar to that of the world level, with two products released: AfSoilgrids1km [96] and AfSoilgrids250m [97]. A considerable effort has been made to rescue soil profile data that were in danger of being lost and that are now compiled into the Africa Soil Profiles database [14, 19-21]. This effort involved two full time positions over a period of nearly five years, plus a number of students assisting in the digitization process and collaboration with six countries, including training sessions. The data rescue in this region has resulted in maps for all properties mentioned in the GlobalSoilMap specifications [11].

Considerable efforts have been made in training and raising technical capacity at locations in seven countries as well as more generally through the yearly Springschool and guest research at ISRIC. These efforts included the compilation and standardization of soil profiles data, the theories and practices of digital soil mapping and even the development of data infrastructures including hardware, software and setting up of data servers. Nowadays, some countries are currently working to develop country level products, based on bottom-up approaches (e.g. Nigeria, Niger, Cameroon, South-Africa, Ghana, Ethiopia), through joining a new GlobalSoilMap consortium and through various bilateral collaborations.

### 6.3 Country-level

#### 6.3.1 Australia

The Australia Soil and Landscape Grids were produced based on the legacy soil data compiled in the National Soil Site Collation database, meeting the GlobalSoilMap specifications on a support of 3x3 arc-seconds [28–29]. There are 13 soil attribute surfaces publically available. The predictions were performed using cubist-kriging. The soil organic carbon content was shown to be distributed according large climatic gradients [127].

#### 6.3.2 United States of America

**Figure 8:**
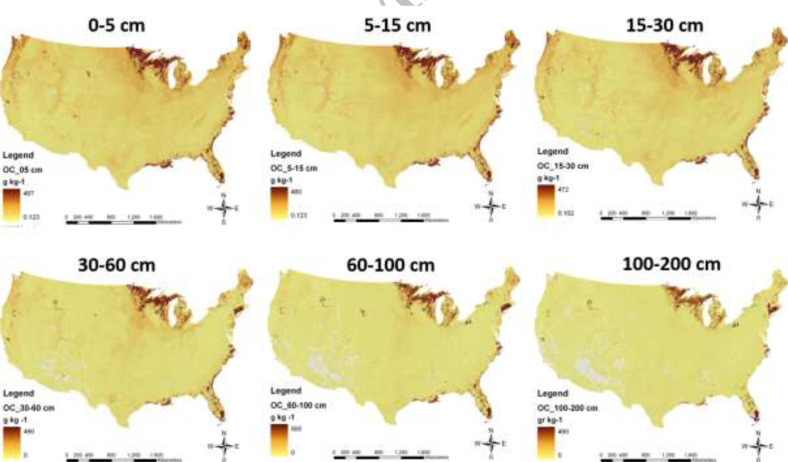
Maps of mean soil organic carbon (g.kg^-^) at the 6 standard depths for continental USA.

The US has produced digital soil maps for the following soil properties: Soil pH; Organic Carbon; Effective Cation Exchange Capacity (ECEC); Soil Bulk Density; Sand, Silt, Clay, Coarse Fragments; Available Water Capacity (AWC); and Rooting Zone Depth, for the standard GlobalSoilMap depths (0-5-15, 15-30, 30-60, 60-100 and 100-200 cm). The predictions are supported by uncertainty measures; the estimated Upper and Lower Limits for each property are considered as the 90% Confidence Limits. Figure 8 shows the Version 0.1 map of soil organic carbon [52].

Here, the highest amounts of organic carbon are found in north central and north east US, mainly associated with forest and south east mainly associated with wetlands. The US product has been produced by mainly using harmonized soil maps from the Digital General Soil Map of the United States or STATSGO2. This is a broad-based inventory of soils at scales 1:250,000, available online at http://websoilsurvey.nrcs.gov

#### 6.3.3 Other countries

Other countries in advanced stages of producing and delivering soil property maps according to the GlobalSoilMap specifications are France [120–123], Denmark [44–45], Scotland [93–94] and Nigeria [70, 72–73]. France recently produced the primary soil properties at 3 arc-second to 3 arc-second resolution [123] and developed an automated to map these properties down to 2-m depth. Several more local trials have been made in regions of some countries [e;g, 37–38, 76–77, 86, 119, 124, 128]. In addition to that, numerous countries have indicated their willingness to join the GlobalSoilMap project.

## 7 Discussion

The number of soil profiles available in national databases is likely underestimated, since responses to our questionnaire from a large number of countries were missed. Moreover, rescuing soil data is an ongoing effort and the number of rescued soil profiles is anticipated to increase substantially. Some countries are involved in long-term soil data rescuing efforts and are far from having completed their programmes. France, for instance, continues an effort to to enrich the national soil database. The year 2015 was chosen for relative comparisons of national soil databases, at the time this paper is published some of them have achieved new data rescue. For instance, data rescuing is still very active in Iran, where about 22,500 new profiles were prepared during 2016 and this process is still ongoing. The Czech Republic indicated that there are about 350,000 scanned soil profiles available from the soil survey of agricultural soils from 1960s. This set of scanned copies is managed by the Research Institute of Soil and Water Conservation (RISWC) and represents a very large potential for improving soil profiles density in the national database of the Czech Republic. Some countries with intensive agriculture, such as Hungary, where national agricultural subsidy systems are linked to compulsory soil tests have produced tremendous amount of soil data with measured coordinates. Unfortunately, no organized data archiving systems exist in these countries to integrate these data and make it available for further use, so these data sources remain only in personal datasets. Making the use of the WoSIS database could contribute to solving this issue.

Other countries (e.g., India, China, Russia, South Korea) have indicated their legacy databases were still under construction. Indeed, most of the these countries are still actively searching for legacy soil information with the potential of many survey reports still to be rescued or retrieved. Therefore, it seems that an enormous potential remains in many countries. The largest country of the world, Russia, undertook many soil surveys in the past, most of which are not yet rescued; this may represent many hundreds of thousands of soil profiles. The global potential for rescuing soil profile data could be in the millions of profiles.

Rescue efforts of legacy soil maps should be pursued. Indeed, in some places of the world this maybe the only available information on soils. This information can be used as default input data to predict a set of soil properties. They can also be used as co-variates for quantitative prediction of these properties. Finally, they are useful to facilitate expert evaluation of digital maps of soil properties. As objectives and concepts of traditional soil mapping varied among countries and evolved with time and advances in knowledge, the issue of harmonization is central if we want to use them for global predictions.

Indeed, very large discrepancies exist among, and even within, national soil databases irrespective of their geographical support (points of polygons). These databases strongly differ in their range of measured soil parameters and in the analytical measurement standards used. Moreover, uniformity in methodology and coverage, albeit existing in some countries, is far from common even among national systems. In view of this situation, it is clear that harmonisation and co-ordination are necessary in order to develop approaches that rescue, harmonize, and curate the existing amount of legacy soil data that is being collected [e.g. 14, 17, 20, 22, 35, 47, 53, 79, 134]. Furthermore, converting results from different analytical protocols to one standard can be done by applying pedotransfer functions, such as listed in [11], which was recently done in the US for pH and bulk density [12–13] and in Africa for available water holding capacity and root zone depth [105].

Nevertheless, soil data rescue efforts have already proven effective in delivering harmonized gridded products of soil properties, with various degrees of resolution and accuracy, and in some cases even covering the world. Numerous countries and institutions have indicated their willingness to join the GlobalSoilMap initiative. A new working group of the International Union of Soil Sciences has been recently created at the end of 2016. As the number of rescued soil data will greatly increase in the near future, it will enable us to deliver consistent high quality products more easily, updated when newly collected data become available. We define a process as ‘bottom-up’ when it comes from a country level action. Most data rescue programmes are based on curating original data from countries and may therefore be considered as ‘bottom-up’. However, the spatial modelling for prediction can be done at the country level, or at the world level as a whole. One of the major expected outcomes of data rescuing is the encouragement and development of country specific bottom-up products (or ‘mixed’ products using ensemble techniques) and capacity development. This should limit the use of generic top-down product approaches, which will nevertheless remain necessary to fill gaps where soil data is missing or lost. We emphasize that GlobalSoilMap is not a static product, but is planned to evolve continuously, as new data or new techniques become available. Legal restrictions related to data property and privacy are serious issues for building an operational worldwide centralized or distributed database of soil profiles and to the complete worldwide and consistent product, useable by global modelers and a host of other users. This is why, when possible, bottom-up approaches in compiling data and producing maps are preferable to top-down.. Another advantage of local modelling is that it may give better results than global modelling which generalizes more the relations between co-variates and soil properties. Indeed, the relative importance of driving factors and co-variates may strongly differ between physiographic areas. This is why utilizing all the data available at country level generally allows to deliver better quality products. It also encourages countries to develop their own capacities, have ownership and support future developments of revised versions of maps representing their mandated country territories. Nevertheless, top-down products, in soil modelling as well as soil data compilation, are certainly useful for GlobalSoilMap as a whole, for a number of reasons:

- They provided early proof of concept,
- They provide a generic product which is complete and covers the globe, being relevant for global users and updateable through country specific possibly collaborative initiatives,
- They allow to fill gaps where soil data is missing or lost,
- They provide geographically continuous data products that are synchronized/harmonized at state/country boundaries and will certainly be useful for final worldwide harmonization,
- They can be combined with country level products, for instance by using ensemble They can be combined with country level products, for instance by using ensemble approaches (refs)

Ultimately, the 90x90 m grid resolution sought by GlobalSoilMap, in addition to providing a seamless product for the global modeling community, is aimed to provide suitable data to a wide variety of communities that makes decisions at various levels from local (field) to national scale and beyond. In this context, the end-user must be informed about the quality of the products, since these maps are predictions which come along with a prediction uncertainty. However, how to properly estimate the prediction uncertainties (and even the uncertainty of the uncertainty) is still a matter of discussion and a question of further research. Several options are described in the GlobalSoilMap specifications [11] and in [129]. Higher level products can be relatively easily validated with lower level data. Furthermore, there is an ongoing effort to better define the accuracy of predictions [51, 78, 86, 93, 129–131] and the sources of uncertainties. Another challenge is how to take into account some large uncertainties, or imprecision in original locations of soil profiles. This is especially relevant and challenging when data of high-resolution are envisioned to be the final products (3 arc-sec). Also, the question of influence on the age of the data rescued has to be solved. Most soil properties are rather stable and have little change (coarse fragments, texture, CEC, soil depth) or change only slowly and steadily over time. However, some properties are rather rapidly changing due to changes in land-use (e.g. pH, soil organic carbon). For instance, a significant change in peat extension in the Netherlands has been recently shown leading to updating soil maps [132]. Moreover, some soil properties may also change very rapidly, at a very local scale, due to farm management practices and thus becoming obsolete for representing the current state of soil. At least, a map of the sampling dates should be added to the GlobalSoilMap specifications. A first draft of this map could be produced rather simply, e.g. by kriging the dates of sampling of the original point data, and would indicate places where data is obviously obsolete.

The issues related to dates not only apply to sampling periods but also to the co-variates used. Obviously, given the long time needed for soil formation, a large number of co-variates used in digital soil mapping do not reflect the reality at some periods of the pedogenesis. Topographic indexes are generally computed using up to date digital terrain models and do not reflect the various steps of geomorphological changes over time. Current climatic data relevance can also be discussed as many soils developed under largely different climatic periods. Indeed as outlined by Grunwald [10] the time factor is much less used in digital soil mapping than other scorpan factors.Ideally, if GlobalSoilMap products are to be used for monitoring, the products should be harmonized to a common date (e.g. 2010), and if funds permit, the products should also be based on newly sampled data. Commonly, most of the current initiatives emphasizing the need for newly sampled data, based on the arguments presented here, focus on collecting new data from topsoil only (e.g. [99–103]). Compared to topsoil sampling, a major advantage of the legacy soil profiles data is that these were sampled to a depth of generally 120 cm or more, providing a more in-depth understanding of soil functions related to various environmental aspects and adequate data for analyses and modelling. Therefore, we recommend that new sampling campaigns sample the full soil profile as well. Indeed, collecting data at different times may be used to assess temporal changes and to perform multi-temporal data updates and queries. Using legacy soil profiles data, Stockmann et al., [133] recently generated products following GlobalSoilMap specifications and incorporating a dynamic component.

## 8 Conclusion

GlobalSoilMap is the first digital soil mapping project having set specifications which have been agreed upon by an international soil science community. Its aim is to cover the entire world with a high resolution grid of predicted key soil properties along with their prediction uncertainties, thereby supporting other scientific disciplines and local management efforts. Significant progress has been achieved since its launch. Data rescue is considered an essential prerequisite to achieve the products and tremendous progress has been made. It is essential that this process be continued; myriads of soil reports and soil maps are certainly still collecting dust on shelves. We encourage soil scientists and librarians to make them available to the soil science community, ideally with digitized georeferenced soil profile data, either at country, continental or world level. Fortunately, numerous countries have indicated their willingness to join the project and continue this important work.

We believe that combining countries and worldwide predictions could lead to a first product completely meeting the GlobalSoilMap specifications by the end of 2020, and that for this purpose both top-down and bottom up approaches are necessary and complementary. Although progress has been made on quantifying the uncertainties of the soil predictions, we believe that further research is still needed on this topic. Ideally, an independent set of validation points, selected through a proper statistical design and possibly from national data holdings, would help to ultimately validate the predictions and to map uncertainties. Providing these uncertainties is essential for the end-users of this product. Also, it would point out those areas in the world where data is too scarce and where new sampling or more data rescue efforts are necessary.
